# Lexicon-enhanced sentiment analysis framework using rule-based classification scheme

**DOI:** 10.1371/journal.pone.0171649

**Published:** 2017-02-23

**Authors:** Muhammad Zubair Asghar, Aurangzeb Khan, Shakeel Ahmad, Maria Qasim, Imran Ali Khan

**Affiliations:** 1 Institute of Computing and Information Technology (ICIT), Gomal University, Dera Ismail Khan, Pakistan; 2 Department of Computer Science, University of Science and Technology, Bannu, Pakistan; 3 Faculty of Computing and Information Technology in Rabigh (FCITR), King Abdul Aziz University (KAU) Saudi Arabia; 4 COMSATS Institute of Information Technology, Abbottabad, Pakistan; Tianjin University, CHINA

## Abstract

With the rapid increase in social networks and blogs, the social media services are increasingly being used by online communities to share their views and experiences about a particular product, policy and event. Due to economic importance of these reviews, there is growing trend of writing user reviews to promote a product. Nowadays, users prefer online blogs and review sites to purchase products. Therefore, user reviews are considered as an important source of information in Sentiment Analysis (SA) applications for decision making. In this work, we exploit the wealth of user reviews, available through the online forums, to analyze the semantic orientation of words by categorizing them into +ive and -ive classes to identify and classify emoticons, modifiers, general-purpose and domain-specific words expressed in the public’s feedback about the products. However, the un-supervised learning approach employed in previous studies is becoming less efficient due to data sparseness, low accuracy due to non-consideration of emoticons, modifiers, and presence of domain specific words, as they may result in inaccurate classification of users’ reviews. Lexicon-enhanced sentiment analysis based on Rule-based classification scheme is an alternative approach for improving sentiment classification of users’ reviews in online communities. In addition to the sentiment terms used in general purpose sentiment analysis, we integrate emoticons, modifiers and domain specific terms to analyze the reviews posted in online communities. To test the effectiveness of the proposed method, we considered users reviews in three domains. The results obtained from different experiments demonstrate that the proposed method overcomes limitations of previous methods and the performance of the sentiment analysis is improved after considering emoticons, modifiers, negations, and domain specific terms when compared to baseline methods.

## Introduction

The Web is a huge repository of facts and opinions available for people around the world about a particular product, service, issue, policy and health-care [1]. With the rapid increase in social media sites, individuals are now relying on user review sites for exchanging their personal information, experiences and knowledge [2].The main focus of the studies in this area has been on issues, such as sentiment detection, sentiment classification at aspect, word, sentence and review levels, opinion spam detection, and context aware sentiment analysis [3]. However, due to the growing interest in computing the exact sentiment of terms within the SA applications, the sentiment classification at word, sentence and review level become an active area of research [4].

In most cases, such large number of information seems unstructured for average internet user. However, it attracted many sentiment analysis researchers towards developing such systems that could assist in analyzing user’s reviews efficiently. User generated reviews poses different challenges due to the specialized nature of the online text. The main challenges faced in developing user centric sentiment analysis applications include: (i) emoticon handling, (ii) low accuracy of the classifier in the sentiment analysis of online content, and (iii) incorrect scoring and classification of domain specific words. Firstly, the emoticon handling issue arises due to insufficient coverage of emoticons expressed by users in their posts. The second challenge is to improve the accuracy of classifier by using unsupervised approach with emphasis on modifiers and negations. The third issue is the limited coverage of domain specific words in the existing general purpose lexicons, such as SentiWordNet (SWN), which assigns incorrect scores to most of the domain specific words and may often result in incorrect scoring and classification of sentiments. The sentiment score of a word is generally dependent on a particular domain and changes when a domain switch occurs.

The aforementioned issues often result in incorrect detection and classification of users’ sentiments expressed in users’ review sites. Therefore, it is an important task to develop a method to detect and analyze the users’ sentiments from online reviews by automatic classification of reviews as positive, negative or neutral.

In this work, we propose a lexicon-enhanced method for improving the sentiment analysis of user generated reviews based on rule-based classification scheme. The main focus is on reducing data sparsity and improving the accuracy of sentiment detection and classification in different domains, effectively reducing the reviews classified as neutral. The proposed method is inspired by the previous studies performed on sentiment analysis of user generated reviews [3, 5]. The previous studies have used un-supervised classification to detect and classify the users’ sentiments expressed by online users into +ive, -ive or neutral classes. However, we use the emoticon classifier, modifier &negation classifier, SWN-based classifier (SWNC) in a sequential way to classify the reviews more accurately. Additionally, we input the text to and domain specific classifier (DSC) to assign accurate sentiment scores to domain specific words, which is one of our major contributions in this work. If the results of SWNC and DSC are identical, then the sentence and review is classified as +ive, -ive or neutral accordingly. However, if there is disagreement between the classifications results of SWNC and DSC, then we consider DSC-based results, because it gives more accurate results with respect to consideration of domain specific words. This assists in improving the performance of sentiment analysis system.

The main contribution of this work over the state of art methods [3, 5] is to handle emoticons, modifiers, negations and domain specific words in an integrated framework. The source code of different modules is available at: https://datadryad.org/resource/doi:10.5061/dryad.p1j71/1

The paper is structured as follows. Section 2 presents literature review. In section3, we describe the proposed method. Experiment design is presented in section 4, which describes the metrics and discussion on obtained results. The final section outlines the work with a discussion on how it can be expanded in future.

## Related work

There are several studies regarding analysis of users’ sentiments from online forums, with focus on classifying the reviews as positive, negative and neutral.

Ferrara and Yang et al. [6], in their work on quantifying the effect of sentiment on information diffusion invested different issues, such as identification of emotions having widespread usage in online text and, whether +ive sentiments are disseminated more than –ive and vice-versa. It was reported that –ive sentiments spread faster than +ive ones and +ive sentiments develops rapidly for highly anticipated events. They identified and classified additional linguistic rules, such as negations, amplifications and emoticons by adopting SentiStrength algorithm. Their approach didn’t address the issue of domain dependent terms, which is one of a major issue in existing sentiment classification systems.

Poria et al. [7] presented a novel mechanism of extracting features from short multimedia-based heterogeneous data, such as textual, audio and visual clips by training the classifier using convolutional multiple kernel learning. For this purpose, they used Deep Convolutional Neural Network (DCNN) model by applying activation values in an inner layer of DCNN. They obtained a performance improvement of about 14% over the baseline methods.

Severyn and Moschitti [8] introduced a convolutional neural network model for performing sentiment analysis of microblogs using deep learning technique. It accurately trains the model without needing any support features. They used an unsupervised neural model for training the seed words which are further subjected to deep learning model. Finally, model is initialized by using pre-trained parameters. Furthermore, supervised learning technique is applied on the Twitter dataset. The system obtained promising results both at phrase level and message level.

In their work on extracting sentiment from text, Taboada et al. [9] developed a Semantic Orientation Calculator (SO-CAL) by using dictionaries of words associated with their sentiment class and score, and includes negation and intensification. The performance of SO-CAL was satisfactory across multiple domains. Moreover, they described the process of dictionary creation and annotation. However, their approach can be enriched by incorporating emoticons and domain specific words for more accurate sentiment classification.

Pensa et al. [10] proposed a concept-level knowledge graph in an integrated framework to represent user behavior on different social media. The active users are tracked by modeling their activities and concepts as well as the relationships with other users. Temporal relationships are also addressed to assist in carrying out temporal analysis. However, incorporation of event detection for automatic detection of hot topics in social networking sites can improve the performance of the system.

Cambria in his recent study [11] reported that emotion recognition and polarity detection are the two basic tasks of affective computing. The former aims at extracting emotion tags and the latter is focused on classifying text into positive and negative classes. The aforementioned tasks are highly co-related and mostly treated in a unified framework for detecting polarity of a sentence and then tagging the sentence with particular emotion category. In many applications, emotion recognition is performed as a subsequent task of sentiment classification.

While working on contextual sentiment analysis for social media genres, Muhammad et el. [12] introduced a lexicon-based sentiment classification method for capturing contextual polarity at local and global levels. The major limitation of lexicon-based approach is incorrect sentiment scoring of opinion words by the existing lexicons, such as SentiWordNet. To address this issue, domain specific vocabulary is introduced to improve the efficacy of sentiment classification.

L. Boratto et al. [13] proposed a technique to detect segments of users for modeling user behavior in advertising. Different data sources are exploited to detect such segments. Firstly, need for user segmentation system is presented to incorporate user preferences successfully, as most of the time is spent by the users on reformulating queries to fulfill their information requirement. Finally, a method is proposed to analyze item description on the basis of user evaluation and extract words in the form of vector notation. The proposed approach is validated by performing experiments on real-world datasets.

Kennedy and Inkpen [14] applied two phase method for measuring the effect of modifiers on classifying the reviews. In the first phase, *General Inquirer* is used to identify positive terms, negations, intensifiers and diminishers. They obtained improved classification results by extending the term-counting technique with context valence shifters. In second phase, machine learning approach, namely Support Vector Machine (SVM) is used by considering unigrams and valence shifter bigrams. They achieved high classification results by using bigram shifters.

The previous studies [6–10] on sentiment analysis used different approaches for analysis, where the supervised learning algorithm [15, 16, 17] is mainly dependent on the availability labeled training dataset. Supervised learning systems are learnt over the labeled training instances to classify the users’ reviews as +ive, -ive or neutral using different features, such as n-grams, part of speech tags and emoticons. Moreover, most of the existing un-supervised approaches [3, 5] do not consider emoticons, modifiers, and domain specific words efficiently. Although such techniques offer satisfactory results for the classification of online content, they pose different challenges. The major challenges are: (i) limited coverage of emoticons, (ii) low accuracy of the classifier in the detection and classification users’ sentiments due to presence of modifiers and negations in online forums,(iii) and inaccurate sentiment classification of domain specific words, as the existing general-purpose lexicons, such as SWN may assign incorrect scores to most of the domain specific words.

The main motivation of this work is the lexicon-based approach suggested by [5], which classifies the reviews based on rule-based technique. They classified the reviews by passing them through different modules, namely, (i) filtering, (ii) subjectivity classification, and (iii) sentiment scoring, to classify the reviews accurately. In a recent work [3], the authors address the issues of sentiment analysis in user reviews, and proposed an effective method of the reviews classification into +ive, -ive, and neutral classes by incorporating slangs using different types of lexicons.

**Fig 1:**
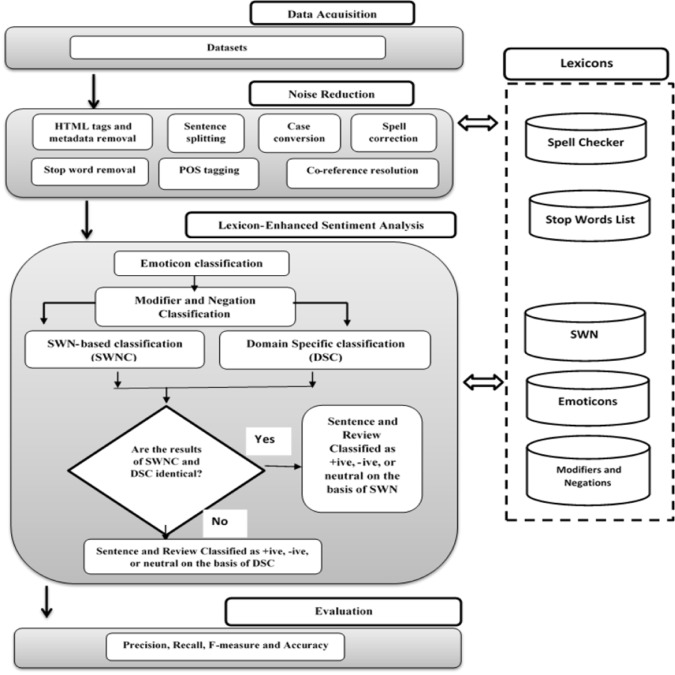
Proposed System.

The proposed system is based on rule-based classification scheme supported by number of repositories, such as SentiWordNet (SWN), emoticon dictionary, modifiers lists and domain specific scoring modules. The major improvement of system over the state of the art methods [3, 5] is in the way it handles emoticons, modifiers, negations and domain specific words in an integrated framework. Our system is capable of automatically detecting and classifying the modifiers, negations, emoticons and domain specific words expressed by users in reviews. That is, we automatically increase, decrease or invert the intensity strength of opinion words by incorporating hand-ranked percentage scale; classify the emoticons by proposing an enhanced rule-based emoticon repository; and finally, opinion words are classified using SWN-based classifier and an improved domain specific classifier. The proposed framework is presented in Fig 1.

## Methods

The proposed method applies different techniques for analyzing and classifying users’ reviews. This involves data acquisition, noise reduction and a rule-based scheme of classification. Data acquisition involves dataset compilation from different resources. Noise reduction steps include: sentence splitting, tokenization, stop word removal, lemmatization, spell correction and co-reference resolution [18]. The proposed technique implements a rule-based scheme using an improved version of emoticon classification [19], enhanced modifier handling [20], sentiment scoring of opinion words using SentiWordNet [21] and an enhanced sentiment classifier using domain specific strategy [22].

The main aim of this work is to enhance the performance of sentiment analysis and resolve the issues of data sparseness and incorrect classification due to use of noisy text, emoticons, modifiers and domain specific words. The basic theme is to reduce noise from the review text by applying different pre-processing steps and process through variant of classifiers. The proposed method is able to test the text from different online forums. The reviews compiled from these sources are used as input items. The method is based on the three major steps: 1) firstly, we acquire the data from different online resources; 2) in next step, the noise reduction is performed by applying different preprocessing techniques to refine the text that can be used for subsequent processing, and 3) finally, different classification techniques are applied to classify the reviews into +ive, -ive or neutral.

### Data acquisition

The data acquisition module is used to compile datasets from user reviews, which serve as input to noise reduction module for filtering the noisy text. For this purpose, we used three user’s reviews datasets, namely: (i)drug (ii) car, and (iii) hotel. Drug review dataset is publically available at: http://ir.cs.georgetown.edu/data/adr/, whereas Car and Hotel reviews are obtained from: https://archive.ics.uci.edu/ml/datasets/OpinRank+Review+Dataset. The reviews are stored in two separate MS-Excel files to compile the testing and training corpuses. This study did not involve any experimental research on humans or animals; hence an approval from an ethics committee was not applicable in this regard. The data collected from the online forums are publicly available data and no personally identifiable information of the forum users were collected or used for this study.

The detail of each dataset is shown in Table 1.

**Table 1:** Sample Datasets.

Datasets	Total # Reviews	Dataset Description
Dataset#1	350	Drug
Dataset#2	273	Car
Dataset#3	412	Hotel

### Noise reduction

In the noise reduction step, noisy text is filtered by applying different preprocessing techniques, including sentence splitting, tokenization, stop word removal, lemmatization, spell correction, case-conversion and anaphoric reference resolution [23]. Moreover, to provide better classification results, unrelated sentences were excluded. For example, in health review dataset, sentences reflecting sympathetic feelings and empathetic encouragements, such as “*Thanks for your suggestion*”, *“wishing your recovery soon”*, or *“I will never leave you alone”*. These comments contain no drug-related information and can be discarded. After noise reduction, the dataset consists of 8,500 reviews with 52% +ive, 42% -ive and 6% neutral reviews.

### Sentiment classification

The rule based classification is used to classify the reviews using set of *“if-then*” rules. The rules are represented in disjunctive normal form (DNF), where *if* clause is called rule antecedent and *then* clause is called rule consequent. The proposed Sentiment Classification Algorithm (SCA) in rule-based framework classifies user reviews by using four classifiers, namely: (i) Emoticon Classifier (EC), (ii) Modifier and Negation Classifier (MNC), (iii) SentiWordNet Classifier (SWNC), and (iv) Domain Specific Classifier (DSC).

The EC is used to classify emoticons on the basis of +ive and –ive emoticon sets. It detects presence or absence of emoticons in a given sentence to classify them as +ive, -ive or neutral. The MNC uses percentage scale based list of +ive and –ive modifiers, stored in two database files; whereas the negation list is a separate text file that includes all possible negation terms. In order to perform sentiment classification of the user’s reviews at word, sentence level and review level, we use SWNC, that uses SentiWordNet (SWN) lexicon to retrieve sentiment score of each word for the classification of reviews. The DSC module is used to perform sentiment classification of such domain specific words, which are, either not present or their sentiment score is not accurately available in SWN.

Algorithm 1 outlines the different steps required for the classification of reviews. Firstly, each review sentence is preprocessed using noise reduction steps, and then different classifiers are applied, as described in the classification module. Finally, the results are generated in the form of +ive, -ive or neutral sentiments at sentence and review level.

Let R denote the set of reviews and W denotes the set of words in each review as:

R={r1,r2,r3…….rn}

W={w1,w2,w3….wn}

We use the following four sentiment classifiers for the final classification of the review sentence.

#### Emoticon Classifier (EC)

Emoticon is a symbolic illustration of mind, mood, emotional state and feelings used by online community [19].Emoticons convey a message more effectively without having dependency on the language and specific domain. They have become vital part of social media chat and public reviews. Therefore, their detection, classification and evaluation have become necessary for the development of efficient sentiment analysis applications.

In this work, emoticon detection is carried out using if-then rules and their classification is based on the set of positive and negative emotions. The proposed module is an enhancement of the work proposed by F.H. Khan et al. [19]. F.H. Khan used a set of 145emoticons, whereas we have extended it to 230; 120 of which are labeled +ive and 110 are labeled as -ive. We hired the services of three human annotators to manually assign polarity class and score to emoticons in our emoticon dictionary. The annotators were informed to assign scores of -1.0 (-ive), 0 (neutral), and +1.0(+ive). The score nearest to the average of the annotators’ scores is computed for each emoticon. Overall inter-annotator agreement is 91.2% with a Kappa (K) score of 0.85, which is quite high.

The partial list of +ive and –ive emoticons are given in Table 2 respectively. The emoticon is labeled as +ive, if it is found in +ive list. If the emoticon is found in the –ive list, then it is labeled as –ive. The emoticon is declared as neutral, if it does not exist in both lists.

**Table 2:** Partial list of positive and negative emoticons.

Emoticon	Meaning	Sentiment Class
:-D	Laughing	Positive
:-)	smile	Positive
o:)-	innocent	Positive
8-)	cool	Positive
:$	Happy blush	Positive
:(	defeated	Negative
:’(	Crying	Negative
:o	shocked	Negative
>(	Grumpy	Negative
(@)	Angry red	Negative
X|	Dead	Negative

Let *E*_*pos*_ *be* a list of positive emoticons and *E*_*neg*_ be a list of negative emoticons associated with each review represented as:

$$E_{pos}=\{list\;of\;positive\;emoticons\}$$

$$E_{neg}=\{list\;of\;negative\;emoticons\}$$

The sentiment score of an emoticon “*e*” is computed as:

$$pol_{score-emo}(e)=\left\{ \begin{array}{c} 1,\;if\;(r\;\epsilon \;R\;⋀\;e\in E_{pos})\\ -1,\;if\;(r\;\epsilon \;R\;⋀e\in E_{neg})\\ 0,\;if\;(r\;\epsilon \;R⋀e\notin E_{pos}⋀e\notin E_{neg})\\ 0,\;if\;(r\;\epsilon \;R⋀e\in E_{pos}⋀e\in E_{neg}) \end{array}\right.$$ (1)

where, *e* denotes the emotion belongs to set of positive and negative emoticons respectively and r is a review from the set of reviews R. The sentiment score of an emoticon “*e”* is a value between 1 and -1, where 1 represents +ive, -1 means –ive and 0 indicates neutral.

#### Modifier and Negation Classifier (MNC)

Modifiers and negations play an important role in the sentiment classification. Detail of the proposed module is described in the following sub-sections.

**Modifier Management:** Modifiers are the words, which enhance or reduce the polarity strength of sentiment words in a sentence, such as: *pretty*, *very*, *slightly*, *somewhat*, *even*, *few*, *too*, *really*, *extremely*, *quite* etc. These words enhance or reduce the polarity strength of an opinion term.

Khan et al. [20], in their work on modifiers, used simple addition and subtraction of constant values. The main issue with this approach is that it does not cover full range of modifiers in particular category. The proposed modifier handling module is an improvement of the work proposed by [20], by using hand-ranked percentage scale to represent variety of modifiers as well as their sentiment scores. We use 75 English modifiers proposed by Benzinger (https://archive.org/stream/intensifiersincu00benz/intensifiersincu00benz_djvu.txt). We assigned a sentiment score to each modifier by using the numeric values proposed by [9, 14]. We converted such numeric scores (e.g. 1, -1, 0.5, -0.5) to respective percentage scales (e.g. +100%, -100%, +50%, -50%) to build of +ive and –ive modifiers lists. The enhancers are +ive, whereas reducers are –ive, as shown in Table 3 and Table 4.

**Table 3:** Partial list of positive modifiers (enhancers).

Modifier	Strength	Modifier	Strength
Completely	+100%	Pretty	+20%
Totally	+70%	Very	+50%
Really	+15%	Too	+45%
Most	+90%	Extremely	+80%
Extraordinarily	+75%		

**Table 4:** Partial list of negative modifiers (reducers).

Modifier	Strength	Modifier	Strength
hardly	-70%	a little	-40%
less	-50%	some	-25%
quite	-20%	a bit	-35%
minor	-30%	slight	-40%
a few	-25%	low	-20%

Let *M*_*pos*_ be a list of positive modifiers and *M*_*neg*_ be a list of negative modifiers represented as:

$$M_{pos}=\left\{list\;of\;positive\;modifiers\right\}$$

$$M_{neg}=\{list\;of\;negative\;modifiers\}$$

If a word is found in a set of positive or negative modifiers, then the polarity of the neighboring opinion word is computed as follows:

$$pol_{score-mod}(w)=\left\{ \begin{array}{l} \left(pol_{score}(w)+(pol_{score}(w)*pm\_score(w_{x}))),\;if\;(r\;\epsilon \;R⋀w_{x}\in M_{pos}\right)\\ \left(pol_{score}(w)+(pol_{score}(w)*nm\_score(w_{y}))),\;if\;(r\;\epsilon \;R⋀w_{y}\in M_{neg}\right) \end{array}\right.$$ (2)

where, *w*_*x*_ and *w*_*y*_ denote the words belonging to a set of +ive and –ive modifiers respectively, w is an opinion word which belongs to a set of words *W*, *r* is a review from a set of reviews *R*, *pm_score(w*_*x*_*)* is the percentage score of +ive modifier and *nm_score(w*_*x*_*)* is the percentage score of -ive modifier retrieved from corresponding modifier dictionaries. The sentiment score of neighboring opinion word is obtained by multiplying the percentage score of modifier by the SWN-based sentiment score of opinion word and then adding it to the SWN-based sentiment score of an opinion word.

For example, in the sentence: *“the medicine is so for very good”*, the modifier *“very”* is enhancing the weight of the adjacent opinion word: *“good”*. Therefore, using Eq 2, the enhanced sentiment score of an opinion word *“good”* is calculated as follows:

*pol*_*score–mod*_("*good*") = *pol*_*score*_(good")+(*pol*_*score*_("good") * *pm_score*("very”) = 0.625+(0.625 x 50%) = 0.625+0.3125 = 0.9375, where, 0.625 is the sentiment score of opinion word, namely “good”, retrieved from SWN and 50% is the strength of positive modifier: “very”, obtained from Table 3, and 0.9375 is modified score of opinion word “good” after the manipulation of modifier.

**Negation Management:** Negation terms, such as: *not*, *never*, *can’t*, *couldn’t*, *didn’t*, and *don’t*, often reverse the polarity of the opinion words in a sentence. For example, the sentences: *“the medicine is effective”* and *“the medicine is not effective”* have different polarities. The first sentence carries positive sentiment, however, in second sentence, the negation term *“not”* reverses the polarity of opinion word *“effective”* from +ive to -ive. Therefore, the negation terms must be properly handled for accurate polarity computation. This work is an adaptation of the work performed by [20] for negation handling. We create a list of negation terms and presence of each word in a sentence is checked.

Let *Neg* be a list of negation words defined as:

Neg={Setofnegationwords}

If a word is found in the negation list, then the polarity of the neighboring opinion word is flipped simply by multiplying the score of opinion word by -1 as follows:

$$pol_{score-neg}(w_{x})=\{(pol_{score}(w_{x})*(-1)),\;if\;((r\;\epsilon \;R⋀(w_{x}-1)\in Neg)$$ (3)

Where *w*_x_ denotes the neighboring opinion word and *w*_x_-1 denotes the preceding word of an opinion word which belongs to a set of negation words *Neg* and *r* is a review from the set of reviews *R*. For example, using Eq 3, the sentiment score of “*not effective*” is computed as follows:

$$=(pol_{score}(effective)*(-1))=0.65\;x\;\text{-}1=\text{-}0.65.$$

In above computation, the polarity score of an opinion word “*effective*” is 0.65, which is obtained from SWN, and after applying negation (Eq 3), it becomes -0.65.

#### SentiWordNet Classifier (SWNC)

This module is used to assign sentiment scores to opinion words using SentiWordNet [24]. Firstly, review document is passed through the NLTK-based (http://www.nltk.org/book/ch05.html) python module which assigns a part of speech tag to each of the word (section 3.1 “noise reduction”). Part-Of-Speech (P.O.S) indicates the property and informativeness of a word [25], thus it is utilized to calculate sentiment scores." After P.O.S tagging, only those terms are considered and searched in SWN, which match the assigned part of speech tag. In this way, terms to be considered are reduced and all senses are not taken into account. If multiple senses belong to a specific term, then the arithmetic mean is computed as follows:

pol_score(w)p=∑=1npol_scorep(i)npos (4)

pol_score(w)n=∑i=1npol_scoren(i)npos (5)

pol_score(w)o=∑i=1npol_scoreo(i)npos (6)

Where “*p*”, *“n”*, and *“o”* denote +ive, -ive and objective scores for particular word (*w*), *n*_*pos*_ represents total number of synsets of the word for corresponding part-of-speech. After computing the mean (average) for different synsets of a word under particular part of speech category, we obtain three scores: +ive, -ive and objective. The final score of the opinion word is calculated as follows:

$$pol_{score-op}\left(w\right)=\left\{ \begin{array}{l} \;pol\_score\;{\left(w\right)}_{p},\;if\;max\left(pol\_score\;{\left(w\right)}_{p},\;pol\_score\;{\left(w\right)}_{n},pol\_score\;{\left(w\right)}_{o}\right)=pol\_score\;{\left(w\right)}_{p}\\ pol\_score\;{\left(w\right)}_{n},\;if\;\text{max}\left(pol\_score\;{\left(w\right)}_{p},\;pol\_score\;{\left(w\right)}_{n},pol\_score\;{\left(w\right)}_{o}\right)=pol\_score\;{\left(w\right)}_{n}\\ \;pol\_score\;{\left(w\right)}_{o},\;else \end{array}\right.$$ (7)

In a given input text, the word “scream” has 6 entries (synsets) in SWN: 3 times as noun and 3 times as verb. If the word “scream” in the input text is noun, then its 3 scores with respect to 3-nouns are represented as:

- scream#3(noun), PosScore = 0.25, NegScore = 0.375, ObjScore = 0.375
- scream#1(noun), PosScore = 0.125, NegScore = 0.0, ObjScore = 0.875
- scream#2(noun), PosScore = 0.0, NegScore = 0.0, ObjScore = 1.0

The aggregated positive, negative and objective scores for the word “scream” are computed using Eq (4), Eq (5) and Eq (6) as follows:

PosScore=0.125,NegScore=0.125,ObjScore=0.75

We select maximum value among the aforementioned three scores, i.e. 0.75, which represents the objective score.

#### Domain Specific Classifier (DSC)

The domain specific words, such as most of words in the health-related domain, have one sentiment class in SWN, whereas, their semantics indicate strong inclination with the other polarity class. If a word’s SWN-based aggregated sentiment score is positive, but its semantics indicate inclination towards negative class as compared to positive ones, we predict the new sentiment class of a word and update the sentiment score accordingly.

The DSC module is used to assign accurate sentiment class and scores to domain specific words. The proposed classifier is inspired from our recent work on domain dependent lexicon generation [26]. In DSC, we adopt their work to predict sentiment class of domain specific words using mutual information concepts and improved feature weighting scheme. However, to assign correct sentiment scores to domain specific words, we propose an alternative strategy based on revised SWN scoring and the manual annotation. The proposed DSC module yields improved results (see results and discussion section) than the comparing methods. It is comprised of two sub-modules, namely (i) Predicting Sentiment Class of a Word and (ii) Modifying Word Sentiment Score.

**Predicting Sentiment Class of a Word:** To predict new sentiment class of a word w with positive or negative class, we adopt Mutual Information (MI) based strategy presented in our previous work [26]. In this strategy, we take two class tags, the positive tag cp and negative tag cn. The sentiment score on SentMI(w, cp) of a word w on class tag cp is computed as:

*Sent*_*MI*_(*w*, *c*_*p*_) of word *w* on class tag *c*_*p*_ is computed as:

$${Sent}_{MI}(w,c_{p})=\alpha MI(w,c_{p})+(1-\alpha )(-MI(w,c_{n}))$$ (8)

Similarly, we compute sentiment score *Sent*_*MI*_ (*w*, *c*_*n*_) as:

$${Sent}_{MI}(w,c_{n})=\alpha MI(w,c_{n})+(1-\alpha )(-MI(w,c_{p}))$$ (9)

Where α is the threshold representing the contributions of *MI*(*w*, *c*_*p*_) and *MI*(*w*, *c*_*n*_), and it ranges from 0 to 1. It is selected after the manual inspection of test sentences for different inputs of unigrams and bigrams. The sentiment score of word *w* for class tag *c* is the linear combination of *MI* with a positive class tag *c*_*p*_ and a negative positive class tag *c*_*n*_. Different values (in the range of 0 to 1) of threshold “alpha” are experimented both for unigrams (e.g., Acute) and bigrams (e.g., Heart-burn). Finally, we come up with conclusion that ideal values of α ranges between {0.3 to 1}. For the unigram, the value of α ranges between {0.3 to 0.7}, and for bigram, it ranges from {0.8 to 1}.

In the next step, we combine *Sent*_*MI*_(*w*,*c*_*p*_) and *Sent*_*MI*_(*w*,*c*_*n*_) as:

$${Sent}_{MI}(w)=\left\{ \begin{array}{l} {Sent}_{MI}(w,c_{p})\;if\;{Sent}_{MI}(w,c_{p})>{Sent}_{MI}(w,c_{n})\\ {Sent}_{MI}(w,c_{n})\;if\;{Sent}_{MI}(w,c_{n})>{Sent}_{MI}(w,c_{p})\\ \;0\;else \end{array}\right.$$ (10)

If *Sent*_*MI*_ *(w*, *cp)>Sent*_*MI*_ *(w*, *cn)*, then the sentiment class of the word *w* is positive, and the accumulative *Sent*_*MI*_ *(w)* sentiment score is +ive. However, if the sentiment of word *w* is negative, then the *Sent*_*MI*_ *(w)* score will be negative. For example, if *Sent*_*MI*_ *(w*, *cn)* = 4 and *Sent*_*MI*_ *(w*, *cp)* = 2.3, then the word *w* tends to be negative and *Sent*_*MI*_ *(w)* = 3. If the value of *Sent*_*MI*_ *(w*, *cp)* is greater than that of the *Sent*_*MI*_ *(w*, *cn)* then the word *w* shows a positive sentiment. Finally, if the aforementioned cases do not stand true, then the sentiment of the word *w* is considered as neutral.

A partial list of unigrams and bigrams along with predicted sentiment class is presented in Table 5.

**Table 5:** Partial list of domain specific terms with predicted sentiment class.

Unigram		Bigram	
Term	Predicted Sentiment Class Using Eq (10)	Term	Predicted Sentiment Class Using Eq (10)
Acute	+ive	Fast acting	+ive
Abrasion	-ive	Heart-burn	- ive
Nausea	-ive	Covering up	+ive
Chronic	-ive	abdominal distension	-ive
Exhaust	-ive	Nervous breakdown	-ive
Deaden	+ive	Heart beat	neu
Relieve	+ive	Bring down	+ive
Able	+ive	Cough up	-ive
Psychotic	-ive	Pulse rate	neu
Insight	+ive	Color blindness	-ive

**Modifying Word Sentiment Score:** When the SWN-based average scores (Eq 4, Eq 5, and Eq 6) represent one sentiment class (positive, negative or neutral) and the *Sent*_*MI*_ *(w)* score of a word (Eq 10) shows another sentiment class of a given word, we modify the sentiment score of such opinion words. Moreover, if a word is not available in SWN then sentiment classification and scoring of such words becomes difficult. This problem can be solved by using a manually crafted scale of +1 to -1 for sentiment scoring of each of the domain specific word.

The proposed method for updating the sentiment score of domain specific sentiment words is an enhancement of the work proposed by [27] and [28]. They used polarity shift method to change the polarity of word from +ive to –ive and vice versa, whereas we have enhanced it for scoring of such words which are not available in SWN by using Manual Scoring Annotation Scheme(Eq 12). This enhancement contributes significantly in the accurate scoring of domain specific words.

The proposed method is formulated using Eq 11 and Eq 12 as follows:

$${\text{pol}}_{\text{score}-\text{ds}}\left(\text{w}\right)=\left\{ \begin{array}{l} \;\text{pos}\_\text{score}\left(\text{w}\right)*\left(-1\right),\left(\text{pos}\_\text{score}\left(\text{w}\right)>neg\_score\left(w\right)\right)˄\left(\text{pos}\_\text{score}\left(\text{w}\right)>obj\_score(w\right)˄\left(\text{SentMI}\left(\text{w}\right)\;\text{is}-\text{ive}\right)\\ \text{neg}\_\text{score}\left(\text{w}\right)*\left(-1\right),\left(\text{neg}\_\text{score}\left(\text{w}\right)>pos\_score(w\right))˄\left(\text{neg}\_\text{score}\left(\text{w}\right)>obj\_score(w\right))˄\left(\text{SentMI}\left(\text{w}\right)\;\text{is}+\text{ive}\right) \end{array}\right.$$ (11)

where *pos_score(w)*, *neg_score(w)*, and *obj_score(w)* are the positive, negative and neutral scores of word *w* in SWN using Eq (4), Eq (5) and Eq (6) respectively; “*Sent*_*MI*_ *(w)* is +ive” shows that the word *w* is labeled +ive using Eq (11) and “*Sent*_*MI*_ *(w)* is -ive” shows that the word *w* is labeled as -ive using Eq (10).

The Eq (11) depicts two possible cases for scoring domain specific words: (i) if a term’s SWN-based sentiment score (Eqs 4, 5, 6) is +ive but its SentMI (w) class is -ive then we invert its +ive sentiment score to –ive, and (ii) if a term’s SWN-based sentiment score (Eqs 4, 5, 6) is -ive but its SentMI (w) class is +ive then we invert its -ive sentiment score to +ive.

**Manual Scoring Annotation Scheme:** The Manual Scoring Annotation Scheme aims at assigning polarity scores to those words which are not available in SWN. Our proposed scheme works as follows: We hired the services of five human annotators who are subject specialists in English language. We provided a list of words generated from our datasets, which are not available in SentiWordNet (SWN). We asked each expert to label the words on a scale of -+0.1 to -+ 1. After performing the manual annotation of entire list, we received five scores for each word. We take average of the five sentiment scores assigned by annotators to each and assign that average score to its corresponding sentiment word. It assists in increasing the scalability of manual evaluation of words not available in SWN.

The Manual Scoring Annotation Scheme is formulated as follows:

$$pol_{score-ds}(w)=\left\{ \begin{array}{l} \;\sum_{i=1}^{5}\{+0.1\;to\;+1\}/5,\;(w\notin SWN)˄(SentMI\;(w)\;is+ive)\\ \sum_{i=1}^{5}\{-0.1\;to\;-1\}/5,\;(w\notin SWN)˄(SentMI\;(w)\;is-ive) \end{array}\right.$$ (12)

The Eq 12 demonstrates that if a word is not available in SWN (*w* ∉ *SWN*) and its SentMI (w) class is +ive, then the score ranges between the average of the five scores of {+0.1 and +1}. If the word is not available in SWN (*w* ∉ *SWN*) and its SentMI (w) class is -ive, then the score ranges between the average of the five scores of {-0.1 and -1} using the aforementioned Manual Scoring Annotation Scheme. For example the word“ *heart-burn”* is not available in SWN and its *Sent*_*MI*_ *(w)*class is –ive. Therefore, the word *“heart-burn”* receives a score of -0.5 using Eq 12. In Table 6, we can observe that the two words, namely “*heart burn*” and “*sore throat*” are not available in SWN, and therefore, their scores are manually crafted by a group of manual annotators in the aforementioned process of Manual Scoring Annotation Scheme.

**Table 6:** Words and their Sentiment coverage.

Term	SentiWordNet Polarity	Modified polarity and score using Eq 11 and Eq 12	Example Sentence
heart-burn	not found	negative(-0.5) (using Eq 12)	I do not like this medicine. It caused **heart-burn.**
sore throat	not found	negative(-0.4) (using Eq 12)	It caused **sore-throat** and blisters on my tongue.
Growth	neutral (1)	negative (-1) (using Eq 11)	The abnormal **growth** on the left shoulder is getting worst.
Relax	Neutral(0.625)	positive(+0.625) (using Eq 11)	It really works well and relaxes my anxiety.
Hospital	Neutral(0.8125)	Negative(-0.8125) (using Eq 11)	I am in **hospital** with server stomach problem.
Clot	neutral (1)	negative (-1) (using Eq 11)	The doctor diagnosed a blood **clot** in the brain.
Dressing	neutral (1)	Positive(+1) (using Eq 11)	The patient’s dressings need to be changed regularly.

#### Sentence and review-level sentiment classification

For each of the sentence in a given review, we compute sentiment score by adding all of the scores of emoticons, modifiers and opinion words present in a sentence. The proposed sentence and review-level sentiment classification is an enhancement of the work proposed by khan et al. [20]. They used limited model of modifiers, whereas we have enhanced it with hand-ranked percentage scale for representing variety of modifiers as well as their sentiment score. Moreover, we have included emoticon and domain specific modules for improving the accuracy of sentiment classification, which we demonstrated in the results and evaluation section.

**SWNC-based sentence level sentiment classification:** Firstly, we classify a sentence using SWNC-based classifier as:

$$sent\_score\_swnc=\sum_{i=1}^{n}\left(pol_{score-emo}(e)+pol_{score-op}(w)+pol_{score-mod}(w)+pol_{score-neg}(w)\right)$$ (13)

Using SWNC scores, we classify the review as:

$$Review\_class\_swnc=\left\{ \begin{array}{c} positve,\;if\;(\sum_{i=1}^{n}sent\_score\_swnc>0)\\ negative,\;if\;(\sum_{i=1}^{n}sent\_score\_swnc<0) \end{array}\right.$$ (14)

The review is classified as +ive, if sum of all scores of sentences is >0 and review is classified as –ive, if sum of all scores of sentences is < 0; otherwise the review is classified as neutral

For example a sentence in health domain is written as: *“It caused* ***slight sore-throat***😐*”*. To perform sentiment classification of this sentence, firstly we classify it using SWNC classifier. For this purpose, we express the polarity scores of opinion words, modifiers, negations and emoticons expressed in the input sentence as follows:

**Emoticon scoring:** There is one emoticon in example sentence, therefore, using Eq 1, polarity score of emoticon = *pol*_*score–emo*_(*e*) = = *pol*_*score–emo*_("😐") = 0, because, according to Eq 1, straight face emoticon is neutral, with score=0.

**Modifier scoring:** Using Eq 2, polarity score of modifier and its associated opinion term is computed as: *pol*_*score–mod*_(*w*) = *pol*_*score–mod*_ ("*slight sore throat*") = *pol*_*score*_(*w*) +(*pol*_*score*_(*w*) * *nm_score*(*w*_*y*_) = 0+[0*(-40%)] = 0. Here, the opinion word “*sore throat*” is not available in SWN, therefore its score is taken as 0, and according to Table 4, the reducer modifier “*slight*” has weightage of -40%. As computed above using Eq 2, we received a score of 0.

**Negation scoring:** using Eq 3, the polarity score of negation term is evaluated as: *pol*_*score–neg*_(*w*) = 0, the 0 shows that there is no negation term, and therefore, negation scoring is not applicable.

**Opinion word scoring:** The scores of the opinion word, namely: “*sore-throat*” is not found in SWN lexicon, and SWNC-based Eq 7 does not assist us in assigning polarity score to such opinion word. Therefore, we assigned 0 to its opinion score as follows: *pol*_*score–op*_(*w*) = 0

Using Eq 12, we compute sentence level score of the given input sentence by combining the aforementioned polarity scores as follows:

$$=\sum_{i=1}^{n}\left(pol_{score-emo}(e)+pol_{score-op}(w)+pol_{score-mod}(w)+pol_{score-neg}(w)\right)=0+0+0+0+0=0$$

The overall sentiment of sentence is neutral, with score = 0.

The major issue with SWNC classifier is that it may result in inaccurate scoring of domain specific words, which may lead to incorrect classification of sentence in multiple domains. For example, “*sore throat*” is a domain specific word, which is not found in SWN. Resultantly, overall score at sentence level for the previous input sentence is neutral (0), which is incorrect.

**DSC-based classification:** To classify such domain specific words more accurately, we further classify a sentence using domain specific classifier (DSC) as:

$$sent\_score\_dsc=\sum_{i=1}^{n}\left(pol_{score-emo}(e)+pol_{score-neg}(w)+pol_{score-mod}(w)+pol_{score-ds}(w)\right)$$ (15)

Finally, using DSC scores, we classify the review as:

$$Review\_class\_dsc=\left\{ \begin{array}{c} positve,\;if\;(\sum_{i=1}^{n}sent\_score\_dsc>0)\\ negative,\;if\;(\sum_{i=1}^{n}sent\_score\_dsc<0) \end{array}\right.$$ (16)

The sentence level score computed in the previous example using SWNC classifier is neutral with sentence level score=**0**, which is incorrect due to incorrect scoring domain specific term: “*sore throat*”. Therefore, we update the polarity scores of such domain specific word(s) using Eq 12, and classify the sentence as:

*pol*_*score–ds*_(*w*) = *pol*_*score–ds*_("*sore throat*") = -0. 4, and using Eq 2, reducer modifier “*slight*” operates on opinion word “*sore throat”* as follows:

$$pol_{score-mod}(w)=pol_{score-mod}("slight\;sore\;throat")=pol_{score}(w)+(pol_{score}(w)*nm\_score(w_{y})=\text{-}0.4+[\text{-}0.4*(\text{-}40\%)]=\text{-}0.24.$$

Now applying Eq 14, we get:

$$=\sum_{i=1}^{n}\left(pol_{score-emo}(e)+pol_{score-neg}(n)+pol_{score-mod}(w)+pol_{score-ds}(w)\right)=0+0+(-0.24)+(-0.4)=-0.64$$

When we compare sentence level score of SWNC classifier (0) with DSC classifier (-0.64), it is observed that the identification and correct scoring of domain specific terms have produced more accurate classification and scoring of entire sentence and helped in reducing the classification anomalies.

If the results of SWNC and DSC are identical, then the review is classified as +ive, -ive or neutral on the basis of SWNC scoring. However, if there is disagreement between the classifications results of SWNC and DSC then we consider DSC-based results, because it gives more accurate results with respect to consideration of domain specific words. This assists in maximizing the efficiency of sentiment classification which was the major limitation in previous studies. As reported in the results and discussion section, the proposed framework performs better than the baseline methods.

#### Proposed Algorithm

An abstract of the steps of the proposed rule-based classification method for implementing the enhanced sentiment analysis are shown as follows:

**Algorithm 1.** Lexicon-Enhanced Sentiment Analysis using rule-based Classification Scheme

**Input:** Users’ reviews

**Output: S**entiment class, sentiment Score

**Begin**

## Read all entries in the corpus

1. **While** (there is sentence in review) **Do**

1.     Perform Preprocessing

2. **if** (a sentence contains opinion word/emoticon))

3.             Subjective Tweet

4.             Call sentiment_scoring(subjective sentence)

5.             Go to step#1 to scan next sentence

6.**else**

7.Objective sentence

8.Go to step#1 to scan next sentence

9.**endif**

10.**If** word found in Emoticon Dictionary

11.             Perform classification using Emoticon Classifier (Eq 1)

12. **If** word found in (Modifier or Negation)Dictionary

13.Perform classification using Modifier and Negation Classifier (Eq 2 and Eq 3)

14. Perform classification using SentiWordNet Classifier (Eq 7)

15. Perform classification using Domain Specific Classifier (Eq 11 and Eq 12)

16. Perform sentiment classification at sentence level (Eq 13 and Eq 15)

17. **End While**

18. Perform SWNC-based classification at review-level using (Eq 14)

19. Perform DSC-based classification at review-level using (Eq 16)

20.

20. Write classification result to file

**End**

## Experiments

We used python and Natural Language Toolkit (NLTK) [29] to implement all of the algorithms presented in Section 3. As described in the data acquisition section, we used multiple datasets to conduct the experiments.

### Results and discussion

In this section, we present and analyze results obtained from the experiments to evaluate the effectiveness of the proposed method by using various evaluation metrics, namely (i) precision, (ii) recall, (iii) F-score, and (iv) accuracy to measure the performance of the proposed technique as follows:

$$Precision\;(p)=\frac{tp}{tp+fp}$$ (17)

$$Recall\;(r)=\frac{tp}{tp+fp}$$ (18)

$$F-measure=\frac{2(p)(r)}{p+r}$$ (19)

$$Accuracy=\frac{tp+tn}{tp+fp+tn+fn}$$ (20)

where, *tp* is the number of true positive reviews correctly classified, *fp* is the number false positive negative reviews incorrectly classified as a positive, *tn* is the number of true negative reviews correctly classified, and *fn* is the number of false positive reviews incorrectly classified as a negative.

The First experiment was carried out to investigate the effect of noise reduction steps applied on the three datasets. Table 7 summarize the results obtained during noise reduction phase by depicting the total number of sentences, number words extracted as incorrect, number of words extracted as correct and the accuracy of the noise reduction steps. Therefore, the proposed noise reductions steps assist in resolving the data sparseness issue efficiently.

**Table 7:** Comparative results obtained for noise reduction phase.

Datasets	Sentences	Incorrect Words Extracted	Correct Words Extracted	Accuracy (%)
Dataset1	8540	1431	1291	90.216
Dataset2	2000	524	462	88.167
Dataset3	2543	874	728	83.295

**Fig 2:**
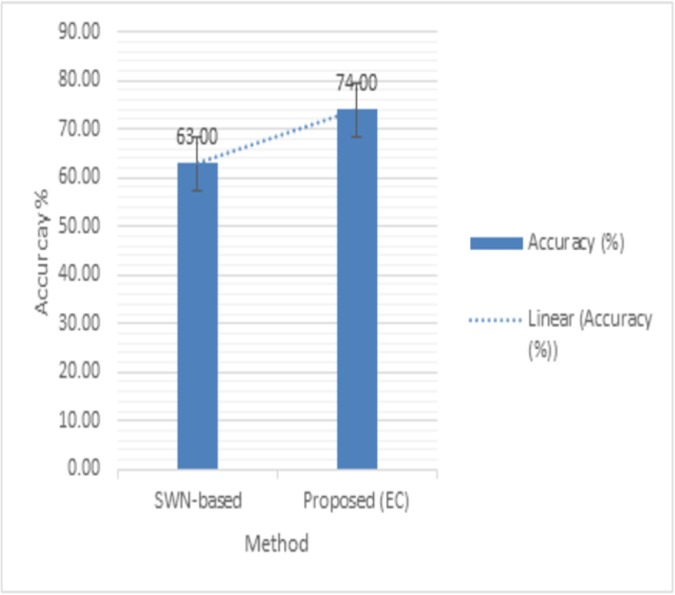
Accuracy results of EC module.

To determine the effect of emoticons in user’s content, we further passed the text through emoticon classifier (EC) module. Our results (Fig 2) revealed that when we incorporated the emoticon handling features in the proposed setup then the accuracy has improved from 63% to 74%.

**Fig 3:**
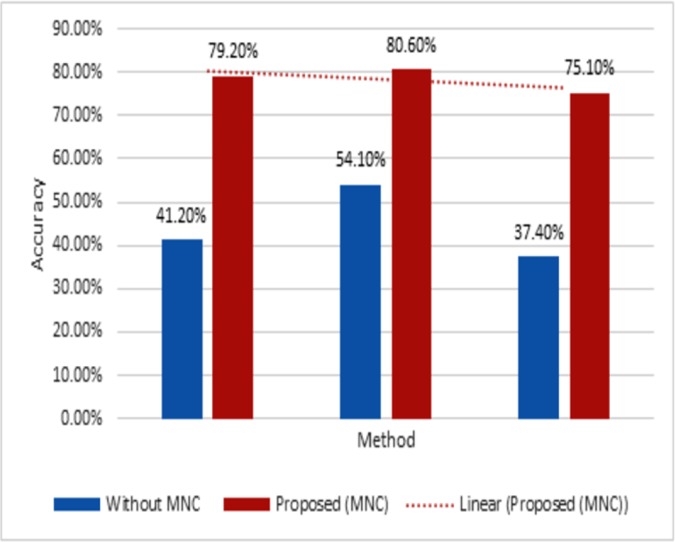
Accuracy results of MNC module.

As described in the section “Modifier and Negation Classifier (MNC)”, modifiers and negations play an important and decisive role in the sentiment classification of user reviews, as they change the polarity of opinion words. In order to evaluate the effectiveness of proposed MNC module, we conducted an experiment on 1951 reviews, split into 14321 sentences. Fig 3 shows that the proposed MNC module yields promising results to classify the input text into +ive, -ive and neutral, effectively increasing the efficiency of sentiment classification of user’s reviews.

**Fig 4:**
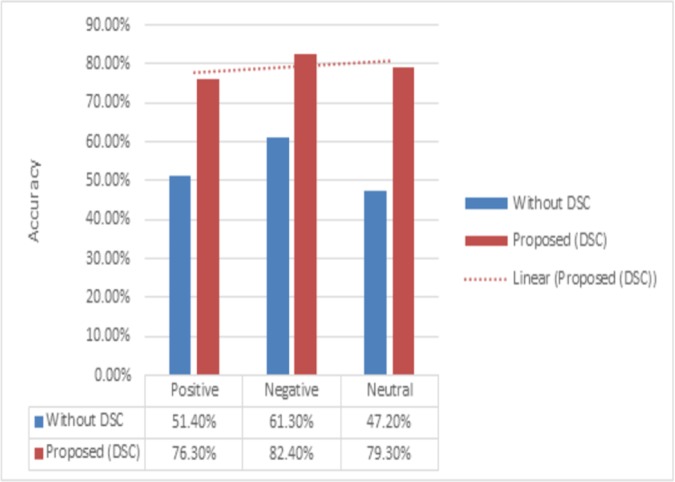
Accuracy results of DSC module.

Fourth experiment was conducted to determine effectiveness of proposed method for the sentiment classification of input text with respect to domain specific words. Due to the specialized nature of certain words, such as words in health-care domain, the sentiment score of such words is not accurately available in existing general-purpose lexicon (SWN). For example, the term “hospital” in SWN has neutral polarity, whereas it is manually annotated as “negative” by medical experts, as most of the times it reflects negative sentiment in our datasets, such as “*I went to hospital due to severe stomach problem*”. Therefore, the term “hospital” is tagged in the negative sentiment class. The comparative results show that when we apply DSC classifier on domain specific words then accuracy of sentiment classification is improved significantly. Fig 4 shows that the proposed method significantly outperforms the non-DSC approach, effectively reducing the number reviews classified as neutral, which was one of challenging task in previous studies.

The final experiment investigates the efficiency of the proposed algorithm on 3 datasets with respect to classification of each review into +ive, -ive or neutral classes. The performance of each of the sub module(classifier) of the proposed framework is evaluated in terms of precision, recall and F-measure. The comparative results show that when all of the classifiers are applied in pipelined way then we achieve promising results. Tables 8, 9 and 10 show that the proposed method significantly outperforms the baseline methods.

**Table 8:** Experimental Results for Dataset1 (P: Precision, R: Recall, F: F-measure).

		Positive			Negative		
Study	Technique	P	R	F	P	R	F
Kalaivani and Shunmuganathan [30]	Supervised (opinion words)	0.80	0.76	0.69	0.74	0.64	0.51
Kundi et al. [5]	Lexicon-based unsupervised (opinion words and emoticons)	0.81	0.79	0.80	0.79	0.82	0.80
Kundi et al.[3]	Lexicon-based Unsupervised (opinion words and emoticons)	0.86	0.78	0.81	0.80	0.82	0.80
Proposed	Lexicon-enhanced-Rule-based (***emoticons*, *opinion words*, *modifiers*, *negations***)	**0.89**	**0.81**	0.79	0.84	0.89	0.81

**Table 9:** Experimental Results for Dataset2 (P: Precision, R:Recall, F:F-measure).

		Positive			Negative		
Study	Technique	P	R	F	P	R	F
Kalaivani and Shunmuganathan [30]	Supervised (opinion words)	0.79	0.63	0.70	0.78	0.71	0.74
Kundi et al. [5]	Lexicon-based unsupervised (opinion words and emoticons)	0.74	0.51	0.60	0.73	0.63	0.67
Kundi et al.[3]	Lexicon-based Unsupervised (opinion words and emoticons)	0.82	0.78	0.79	0.75	0.73	0.73
Proposed	Lexicon-enhanced-Rule-based (***emoticons*, *opinion words*, *modifiers*, *negations***)	**0.83**	**0.94**	**0.85**	0.79	0.77	0.74

**Table 10:** Experimental Results for Dataset3 (P: Precision, R: Recall, F:F-measure).

		Positive			Negative		
Study	Technique	P	R	F	P	R	F
Kalaivani and Shunmuganathan [30]	Supervised (opinion words)	0.52	0.71	0.59	0.83	0.76	0.79
Kundi et al. [5]	Lexicon-based unsupervised (opinion words and emoticons)	0.74	**0.95**	0.65	0.72	**0.96**	0.82
Kundi et al.[3]	Lexicon-based Unsupervised (opinion words and emoticons)	0.71	0.85	0.77	0.77	0.77	0.77
Proposed	Lexicon-enhanced-Rule-based (***emoticons*, *opinion words*, *modifiers*, *negations***)	**0.81**	0.93	**0.88**	**0.84**	0.74	**0.84**

### Descriptive statistics on review data

In Table 11, we present statistics based on the review data obtained from publically available datasets. We randomly sampled a set of 350, 373 and 412 reviews from drug, car and hotel domains. The three datasets are composed of approximately 3,500 sentences and 52,000 tokens. The average length of review is almost same in all of the three domains, while drug reviews (10.61 sentences/review) are slightly lengthy as compared to other two domains (9.56 and 10.21 sentences/review). Similarly, the average length of a sentence is same with 18.47, 18.58 and 18.38 tokens per sentence. The standard deviation of sentences in a review of drug dataset is low as compared to other two datasets. The smallest review in all datasets are comprised of single sentence and the smallest sentences composed of a single token only. The standard deviation of tokens in a sentence of car dataset is low as compared to other two datasets. The average number of stop words per sentence is between and 3 and 4. The average number of negations per sentence is between and 1 and 2, and the average number of modifiers per sentence is between and 2 and 3.

**Table 11:** Descriptive statistics of the proposed system on three datasets.

Statistic	Drug	Car	Hotel
Reviews	350	373	412
Sentences	3525	3553	3561
Average Length (sentence/review)	10.61	9.56	10.21
Std. Dev sentence/review	8.06	12.14	11.21
Min. sentence/review	1.00	1.00	1.00
Max. sentence/review	35.00	19.00	41.00
Total no. of tokens	52041	52231	52482
Average tokens (tokens/sentence)	18.47	18.58	18.38
std. Dev tokens/sentence	10.21	10.04	10.43
Min. tokens/sentence	1.00	1.00	1.00
Max. tokens/sentence	82.00	79.00	88.00
avg. stop words/sentence	4.00	3.00	4.00
avg. negations/sentence	2.00	1.00	1.00
avg. modifiers/sentence	2.00	2.00	3.00

## Conclusions and future work

This article presents the results of applying an improved method based on four way rule-based classification scheme to detect and classify sentiments expressed by users in online discussion forums. The proposed method is comprised of following modules: 1) Acquire set of reviews that mention user reviews about different products; 2) Apply noise reduction steps; 3) Use emoticon classifier to detect and score the emoticons expressed in reviews; 4) Perform classification and scoring of modifiers and negations using a set of positive and negative modifiers and negation list; 5) Apply sentiment classification of words using SentiWordNet-based classifier; 6) Detect the domain specific words and label them with correct sentiment class and score, and; 7) Perform sentiment classification of reviews at sentence and review level.

This approach provides an integrated rule-based framework for sentiment analysis with emphasis on emoticon classification, proper management of modifiers and negations, performs SWN-based sentiment classification, and improves the classification accuracy and enhances the performance of sentiment classification for domain specific words using domain specific classifier. We obtained classification results with improved accuracy, precision, recall and F-measure as compared to comparing methods. The proposed method is quite generalized and can classify the sentiments in cross domain.

A possible limitation of this method regarding its classification efficacy for domain specific words is the need for automatic classification and scoring of words. In order to reduce the amount of manpower required for manual scoring of domain specific words, the possibility of using auto-scoring techniques should be investigated. Another possible way of improving and extending the technique is by exploiting semantic and contextual features to classify the sentiments efficiently. Another interesting research direction would be to study the sentiments of online users in microblogging sites, such as Twitter, in streaming fashion.

## Supporting information

S1 File (RAR)
